# The Novel Approach to Enhance Seed Security: Dual Anti-Counterfeiting Methods Applied on Tobacco Pelleted Seeds

**DOI:** 10.1371/journal.pone.0057274

**Published:** 2013-02-28

**Authors:** Yajing Guan, Jianchen Wang, Yixin Tian, Weimin Hu, Liwei Zhu, Shuijin Zhu, Jin Hu

**Affiliations:** Seed Science Center, College of Agriculture and Biotechnology, Zhejiang University, Hangzhou, P.R. China; United States Department of Agriculture, United States of America

## Abstract

Seed security is of prime importance for agriculture. To protect true seeds from being faked, more secure dual anti-counterfeiting technologies for tobacco (*Nicotiana tabacum* L.) pelleted seed were developed in this paper. Fluorescein (FR), rhodamine B (RB), and magnetic powder (MP) were used as anti-counterfeiting labels. According to their different properties and the special seed pelleting process, four dual-labeling treatments were conducted for two tobacco varieties, MS Yunyan85 (MSYY85) and Honghua Dajinyuan (HHDJY). Then the seed germination and seedling growth status were investigated, and the fluorescence in cracked pellets and developing seedlings was observed under different excitation lights. The results showed that FR, RB, and MP had no negative effects on the germination, seedling growth, and MDA content of the pelleted seeds, and even some treatments significantly enhanced seedling dry weight, vigor index, and shoot height in MS YY85, and increased SOD activity and chlorophyll content in HHDJY as compared to the control. In addition, the cotyledon tip of seedlings treated with FR and MP together represented bright green fluorescence under illumination of blue light (478 nm). And the seedling cotyledon vein treated with RB and MP together showed red fluorescence under green light (546 nm). All seeds pelleted with magnetic powder of proper concentration could be attracted by a magnet. Thus, it indicated that those new dual-labeling methods that fluorescent compound and magnetic powder simultaneously applied in the same seed pellets definitely improved anti-counterfeiting technology and enhanced the seed security. This technology will ensure that high quality seed will be used in the crop production.

## Introduction

High quality seed is one of important factors that determine the potential for rapid and homogenous germination, which providing both economical and environmental benefits in agriculture and horticulture, as they allow for higher degrees of automation, easier weed control and a reduction of disease pressure in the field [1]. Pelleting is a kind of seed postharvest processing technologies [2] and has been used wildly to enhance the seed quality through providing seeds a suitable microenvironment for their fast germination and better growth. Most of small seeds such as tobacco, flower and vegetable seeds, usually have high-value and large-profit. It is very common and useful for increasing the size of small or fine seeds though pelleting technology to produce bigger, rounder, smoother and more uniformly-sized coated products [3]. These products will be more suitable for precise placement in the field and greenhouse. Moreover, improved planting placement will increase final-stand establishment, crop uniformity, and decrease seed and production costs [4]. However, on account of the utter change of seed original shape during pelleting craft, the identification of true seeds from fake ones becomes more difficult. In addition, the high-value and large-profit of pelleted seeds made them more likely to be forged.

The fake or counterfeiting seeds pretending to high quality seeds will seriously ruin the crop yield and quality in agriculture production [5], [6]. How to ensure seed security has caused more and more attention. Most of seed enterprises do their best to eliminate fake seeds in seed market, however, it is definitely difficult to be accomplished just though seed anti-counterfeiting package technologies [7], [8]. Seed is a special kind of business product. The authenticity of most species could not be discerned easily by the naked eyes. Sometimes, it is also pretty difficult to confirm the true seed sources [5]. Therefore, new anti-counterfeiting methods for seeds, especially for pelleted seeds, are needed urgently.

There are many kinds of fluorescent compounds which had been widely applied in clinical diagnosis, food and biology in recent years, based on their better characteristics of easy operation and high stability [9]. Some of them also have been used for plant or seed study. For instance, Shi *et al.* [10] successfully mimicked the performance of water moving into the wheat kernels through fluorescent dyes. Van *et al.* [11] used fluorescent indicator HPTS (8-hydroxy, 3, 6-three acid pyrene) to trace the route of nutrients into the pea seed coat. Present study emphasized how to label pelleted seed directly through fluorescent marker to confirm seed authenticity. For utility, the fluorescent material for labeling seed should be able to enter the seed or absorb by radical into the whole seedling. Thereby the fluorescent compounds with systemic activities were selected for this study due to their better movement abilities in plant. Lipophilicity, solubility, molecular weight and so on are important parameters to predict the systemic activity of organic compounds in plant tissues [12]. Lipophilicity is measured by the octanol/water partition coefficient (LogK_ow_) and is related to the ability of organic compounds to cross the plasma membrane. Moderately lipophilic materials permeate membranes rapidly, while hydrophilic compounds are unable to pass through the membrane readily, as well as compounds with high lipophilicity. Thus compounds with systemic activity have octanol/water partition coefficient in the range LogK_ow_ of −0.5 to 3.5 [13]. Better systemic activity in roots and shoots was determined for compounds with intermediate polarity with LogK_ow_ between 1.5 and 3.5 [13], [14]. Water solubility of chemicals does not control membrane permeability. However, it is important that compounds diffuse to the plant surface in a soluble state [15]. Membrane permeation is also related to molecular weight of compounds. The effective molecular weight for agrochemicals is about 300 g/mol [13].

Fluorescein (FR) and rhodamine B (RB) are common fluorescent dyes. The LogK_ow_ and the molecular weight of FR are 3.35 and 323 g/mol, and those of RB are 1.85 and 479 g/mol, respectively, so both of them belong to systemic material. FR had been commonly used to detect ophthalmic lesions in humans and animals, such as corneal ulcer [16]. Noga and udomkusonsri [17] also found that FR could be used as a highly rapid, efficient and sensitive indicator of skin damage in fish. FR could improve the root performance and promote plant growth and development under lower concentration [18] and the similar results were also found by Li *et al.* [19]. Those researches predicted that FR was relatively safe to animal and plant. RB is a water-soluble systematic fluorescent compound which has better light stability and higher fluorescence quantum yields compared with other fluorescent dyes. FR and RB have been widely used in biology, environmental chemistry, single molecule detection, fluorescence tagging and so on [20], [21].

Moreover, magnetic powder (MP) also could be used as a kind of seed label. Some reports indicated that magnetic suction precision sowing could be achieved by adding magnetic powder into seed coating agent [22]. It meant that the seeds coated with magnetic powder could be detected or selected out by some special machines such as magnetic precision drilling. Meanwhile, magnetic field treatment could accelerate seed germination and seedling growth, and enhance the absorption capacity of fertilizer and water and photosynthetic [23]. Liu *et al.* [22] found that after pelleting with 20% (W/W) magnetic powder and magnetizing with 100 mT magnetic field strength, rape seed germination and growth were evidently improved.

In the last two years, the single fluorescent anti-counterfeiting methods had been reported on some naked seeds [24], [25]. However, the combined application of fluorescent dye and magnetic powder on pelleted seed as dual anti-counterfeiting labels had not been reported up to now. For single-label anti-counterfeiting methods, the only marker makes them easy to be forged. For dual-label pelleting technologies, two markers will increase the difficulty of imitation; in addition, appropriate ratio of magnetic powder could accelerate seed germination and seedling growth [23]. Therefore, the present investigation was conducted to provide some new approaches by using dual anti-counterfeiting labels for enhancing the security of pelleted seed and for further ensuring the seed quality. Meanwhile, some physiological parameters were also determined to test the effect of this novel method on seed germination and seedling growth.

## Materials and Methods

### Materials

The seeds of tobacco (*Nicotiana tabacum* L.) cv. MS Yunyan 85 (MSYY85) and Honghua Dajinyuan (HHDJY), coating agent (talc and bentonite) and the adhesive from Yunnan Provincial Academy of Tobacco Agricultural Sciences, China, were used. Fluorescein (FR) and rhodamine B (RB) was obtained from Advanced Technology and Industrial Co. Ltd., Shenzhen, China, and magnetic powder (MP) (99% purity and 250 meshes) was purchased from Shanghai CNPC Powder Material Co. Ltd., Shanghai, China.

### Preparation of the Tobacco Seed Pellets with Dual-labels

During pelleting process, tobacco seeds were first coated with pure water and bentonite in a cyclic alternating pattern until the seed size reached to 1.00∼1.25 mm-diameter (5∼8 ml water per gram of naked tobacco seeds sprayed in total), then a second layer of talc and adhesive solution were supplied cyclically until the seed size was 1.60∼1.80 mm-diameter. The first layer that directly covered the whole seed was bentonite, the second layer close to bentonite mainly consisted of talc. Pelleting treatments were prepared for each variety as follows:

- Ck: 2 g seeds pelleted with 15 g bentonite and 84 g talc as a control.
- T1: 2 g seeds pelleted with 15 g bentonite and 84 g powder mixture which consisted of 79.6 g talc, 0.2 g fluorescein and 4.2 g magnetic powder. (FR and MP dual-labels)
- T2: 2 g seeds pelleted with 15 g bentonite and 84 g powder mixture which consisted of 77.08 g talc, 0.2 g fluorescein and 6.72 g magnetic powder. (FR and MP dual-labels)
- T3: 2 g seeds pelleted with 15 g bentonite and 84 g powder mixture which consisted of 79.8 g talc and 4.2 g magnetic powder. Meanwhile, a 2.0 mg/ml of rhodamine B solution was sprayed in place of pure water when the seed was coated with bentonite. (RB and MP dual-labels)
- T4: 2 g seeds pelleted with 15 g bentonite and 84 g powder mixture which consisted of 77.28 g talc and 6.72 g magnetic powder. Meanwhile, a 2.0 mg/ml of rhodamine B solution was sprayed in place of pure water when the seed was coated with bentonite. (RB and MP dual-labels)

FR only existed in the second layer and was applied at a rate of 1 g per 10 g of naked seeds in the T1 and T2 treatments. MP only existed in the second layer and was applied at a rate of 5 g per 100 g of mixture powder in the T1 and T3 treatments, and at a rate of 8 g per 100 g of mixture powder in the T2 and T4 treatments. Meanwhile, RB only existed in the first layers in the T3 and T4 treatments. All seeds were pelleted by a minitype coater “BY300A” (Shanghai, China) and then were air-dried for 2 days at room temperature.

### Investigation of Seed Germination and Physiological Changes in Tobacco Seedlings

Following pelleting, seed germination test was carried out. 100 seeds were placed in a 9 cm-diameter Petri dish with 3 layers of water-moistened blotters, and three replications of 100 seeds each for each treatment were used. Then, seeds were incubated in a growth chamber (DGX-800E, made in Safe Experiment Instrument Factory, China) with 250 µmol⋅m^−2^⋅s^−1^ light intensity and an alternating cycle of 8 h light at 30°C and 16 h darkness at 20°C for 16 days [26].

#### Seed germination and seedling growth

The germinated seeds were recorded daily for 16 days. Then germination energy (the percentage of normal germinated seeds in all tested seeds at the 7^th^ day for tobacco) and germination percentage (the percentage of normal germinated seeds in all tested seeds at the end of the full test) were calculated at 7 and 16 d, respectively [26]. Germination index (GI) and vigor index (VI) were calculated according to Hu *et al*. [27]: GI = ∑(*G_t_*/*T_t_*), where *G_t_* is the number of the new germination seeds in times of *T_t_*; VI = GI × seedling dry weight. In addition, root length and shoot height were manually measured with a ruler, and seedling dry weight was determined after drying at 80°C for 24 h [28]. Those measurements were made on thirty randomly selected normal tobacco seedlings for each replicate [29].

#### Protective enzymes and MDA content

Superoxidase dismutase (SOD) activity in seedlings was determined by the method described by Cao *et al.* [30]. The malondialdehyde (MDA) content in seedling was measured using thiobarbituric acid reaction method [31]. All measurements mentioned above were made after seed germination for 16 d. An absorbance change of 0.01 in one minute was used as 1 unit of the enzyme activity (U): U/gFW·min.

#### Chlorophyll products assay

After germination for 16 d, fresh seedlings were chopped fine and weighted 0.2 g by an analytical balance, then homogenized in a homogenizer with the addition of 10 ml of 95% ethanol. A primary ethanol extract containing all chloroplast pigments was obtained in this way. The extract was then centrifuged at 5000 r/min for 10 min. Since the concentration of pigments was in most cases too great for reading to be performed on a spectrophotometer, the obtained extract was diluted by adding 4 ml of 95% ethanol per ml of extract. The absorbance of the extract produced in this way was measured on a spectrophotometer at 470, 649 and 665 nm. Chlorophyll a (Chl-a), chlorophyll b (Chl-b), carotenoids (Cx-c), and total chlorophyll (Chl a+b) contents were calculated according to Wang *et al.* [32].

### Fluorescence Detection

After pelleted seeds germinated for 7 and 16 days, fluorescence existing in seedlings was observed by fluorescence microscope (Leica MZ16FA) and their micrographs were taken by the camera (Leica DFC42C). The photos of the fluorescence in cracked pellets excited by green (546 nm) and blue light (495 nm) were also been taken.

### Statistical Analysis

Statistical analysis of all data was conducted by means of analysis of variance (ANOVA) using Statistical Analysis System (SAS) software (version 8.0). Fisher’s least significant difference (LSD) tests (α = 0.05) were adopted for multiple comparison. The percentage data were arcsin - transformed before analysis according to 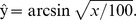


## Results

### Effects of Dual-labeling Treatments with Fluorescent Material and Magnetic Powder on Germination and Seedling Growth of Tobacco Pelleted Seed

There were no significant differences in germination energy, germination percentage, germination index and root length among the control, T1, T2, T3 and T4 irrespective of tobacco varieties (Table 1 and 2). All treatments improved the seedling dry weight of MSYY85, and the T2 and T3 reached a significant level. Meanwhile, the vigor index of T2, the shoot height of T1 and T2 were also significantly increased compared with the control. However for HHDJY, the seedling dry weight and vigor index of the four dual-labeling treatments had no significant differences with the control.

**Table 1:** Effects of dual-labeling methods on tobacco pelleted seed germination.

Variety	Treatment**	Germination energy (%)	Germination percentage (%)	Germination index
MSYY85	Ck	95.1±4.2a∗	98.9±1.1a	16.46±0.7a
	T1	97.4±0.0a	99.8±0.2a	17.48±0.2a
	T2	97.0±4.1a	98.3±2.9a	17.86±0.6a
	T3	98.4±2.7a	98.4±2.7a	17.31±0.5a
	T4	95.0±4.6a	98.6±2.4a	16.47±1.1a
HHDJY	Ck	96.2±3.3a	98.1±3.3a	16.80±0.7a
	T1	96.2±5.2a	98.4±2.2a	16.90±1.0a
	T2	97.1±4.9a	97.1±4.9a	16.94±1.2a
	T3	94.5±4.7a	98.6±1.3a	16.69±0.5a
	T4	95.5±4.6a	97.8±3.9a	16.63±0.7a

* Significant difference (α = 0.05, LSD) among treatments within the same variety.

** **Ck**: 2 g seeds pelleted with 15 g bentonite and 84 g talc as a control; **T1**: 2 g seeds pelleted with 15 g bentonite and 84 g powder mixture which consisted of 79.6 g talc, 0.2 g Fluorescein and 4.2 g magnetic powder (FR and MP dual-labels); **T2**: 2 g seeds pelleted with 15 g bentonite and 84 g powder mixture which consisted of 77.08 g talc, 0.2 g Fluorescein and 6.72 g magnetic powder (FR and MP dual-labels); **T3**: 2 g seeds pelleted with 15 g bentonite and 84 g powder mixture which consisted of 79.8 g talc and 4.2 g magnetic powder. Meanwhile, a 2.0 mg/ml of rhodamine B solution was sprayed in place of water when the seed was coated with bentonite (RB and MP dual-labels); **T4**: 2 g seeds pelleted with 15 g bentonite and 84 g powder mixture which consisted of 77.28 g talc and 6.72 g magnetic powder. Meanwhile, a 2.0 mg/ml of rhodamine B solution was sprayed in place of water when the seed was coated with bentonite (RB and MP dual-labels).

**Table 2:** Effects of dual-labeling methods on seedling growth of tobacco pelleted seeds.

Variety	Treatment**	Root length (mm)	Shoot height (mm)	Dry weight (mg/100 plants)	Vigor index
MSYY85	Ck	9.17±0.42a^∗^	9.33±0.54ab	6.71±0.24b	0.110±0.008b
	T1	10.17±0.65a	10.55±0.61a	7.00±0.18ab	0.123±0.005ab
	T2	10.81±0.45a	10.45±0.15a	8.03±0.69a	0.143±0.017a
	T3	10.86±1.45a	8.88±0.47b	7.92±0.68a	0.138±0.014ab
	T4	11.34±1.09a	9.08±0.53b	7.53±0.21ab	0.123±0.009ab
HHDJY	Ck	12.81±1.15a	8.43±0.56a	7.82±0.62a	0.130±0.005a
	T1	14.34±1.13a	8.58±0.87a	7.52±0.51a	0.127±0.016a
	T2	14.51±1.13a	8.71±0.95a	7.91±0.57a	0.133±0.008a
	T3	14.49±1.07a	8.17±0.25a	7.83±0.02a	0.131±0.004a
	T4	13.96±0.44a	8.36±0.77a	8.50±0.24a	0.142±0.010a

* Significant difference (α = 0.05, LSD) among treatments within the same variety.

** For other explanation see Table 1.

### Effects of Dual-labeling Treatments with Fluorescent Material and Magnetic Powder on SOD Activity and Contents of MDA and Chlorophyll of Tobacco Pelleted Seed

**Figure 1:**
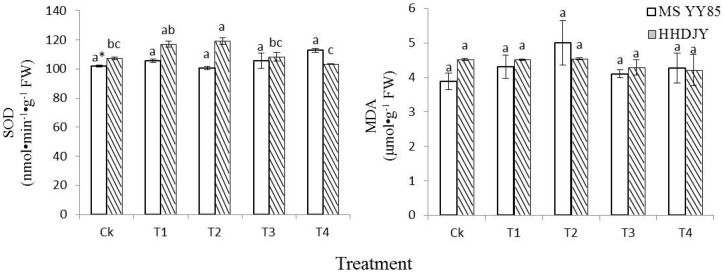
Effects of dual-labeling methods on superoxidase dismutase (SOD) activity and malondialdehyde (MDA) content of tobacco seedling. *significant difference (α = 0.05, LSD) among treatments within the same variety. Error bars represent ±S.E.

**Figure 2:**
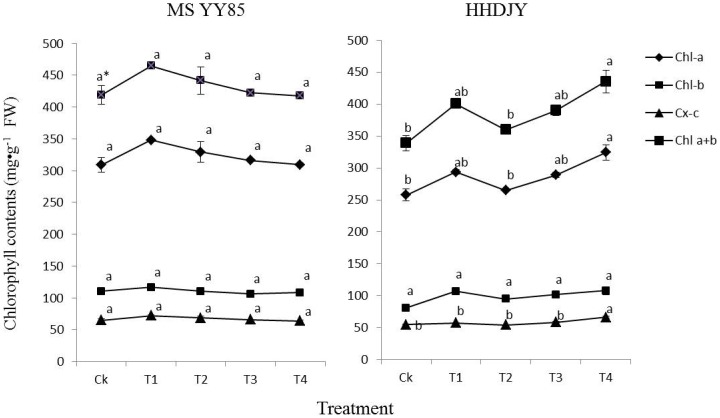
Effects of dual-labeling methods on chlorophyll a (Chl-a), chlorophyll b (Chl-b), total chlorophyll content (Chl a+b) and carotenoids (Cx-c) contents in tobacco seedling. *significant difference (α = 0.05, LSD) among treatments within the same variety. Error bars represent ±S.E.

For MSYY85, there were no significant differences in SOD activity and the contents of MDA, chlorophyll a, chlorophyll b, carotenoids and total chlorophyll among the control and the four pelleting treatments (Figure 1 and Figure 2). However for HHDJY, the SOD activity of T2 was significantly higher than that of the control (Figure 1). The MDA contents of four treatments had no significant differences from the control. Meanwhile, after treated with T4, the contents of chlorophyll a, carotenoids and total chlorophyll in seedling were increased significantly compared with the control (Figure 2).

### Effects of Dual-labeling Treatments with Fluorescent Material and Magnetic Powder on Fluorescence of Pelleted Seed and Seedling

**Figure 3:**
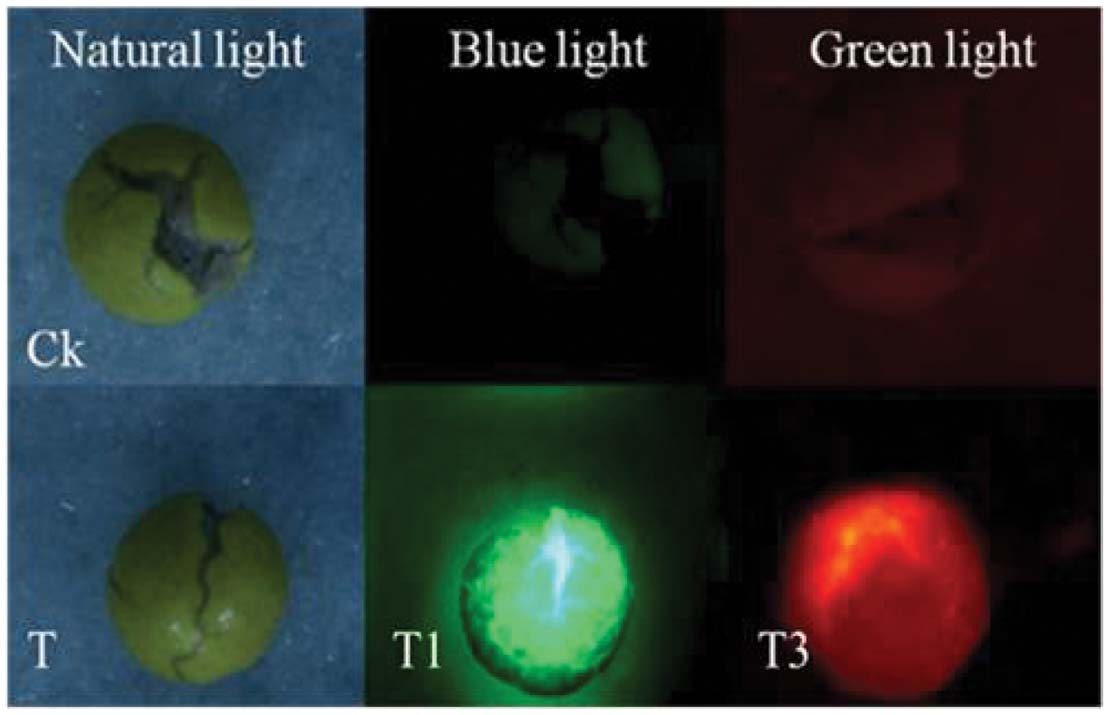
Fluorescence in cracked seeds of MS YY85 pelleted with dual-labeling method under illumination of different lights. Ck: the top row, the control pellets treated without fluorescent materials and magnetic powder (×7); three columns from left to right: under natural light, blue light and green light, respectively; T: Seeds pelleted with fluorescent materials and magnetic powder under natural light; T1: 2 g seeds pelleted with 15 g bentonite and 84 g powder mixture which consisted of 79.6 g talc, 0.2 g fluorescein and 4.2 g magnetic powder (FR and MP dual-labels); T3: 2 g seeds pelleted with 15 g bentonite and 84 g powder mixture which consisted of 79.8 g talc and 4.2 g magnetic powder. Meanwhile, a 2.0 mg/ml of rhodamine B solution was sprayed in place of water when the seed was coated with bentonite (RB and MP dual-labels).

When cracked after adding a drop of water, the pellets of MSYY85 treated with FR and magnetic powder together showed bright green fluorescence under blue light excitation, and the ones treated with RB and magnetic powder together exhibited red fluorescence under green light illumination in comparison of control (Figure 3). However there were no difference between the treated seed and the control seed under natural light.

**Figure 4:**
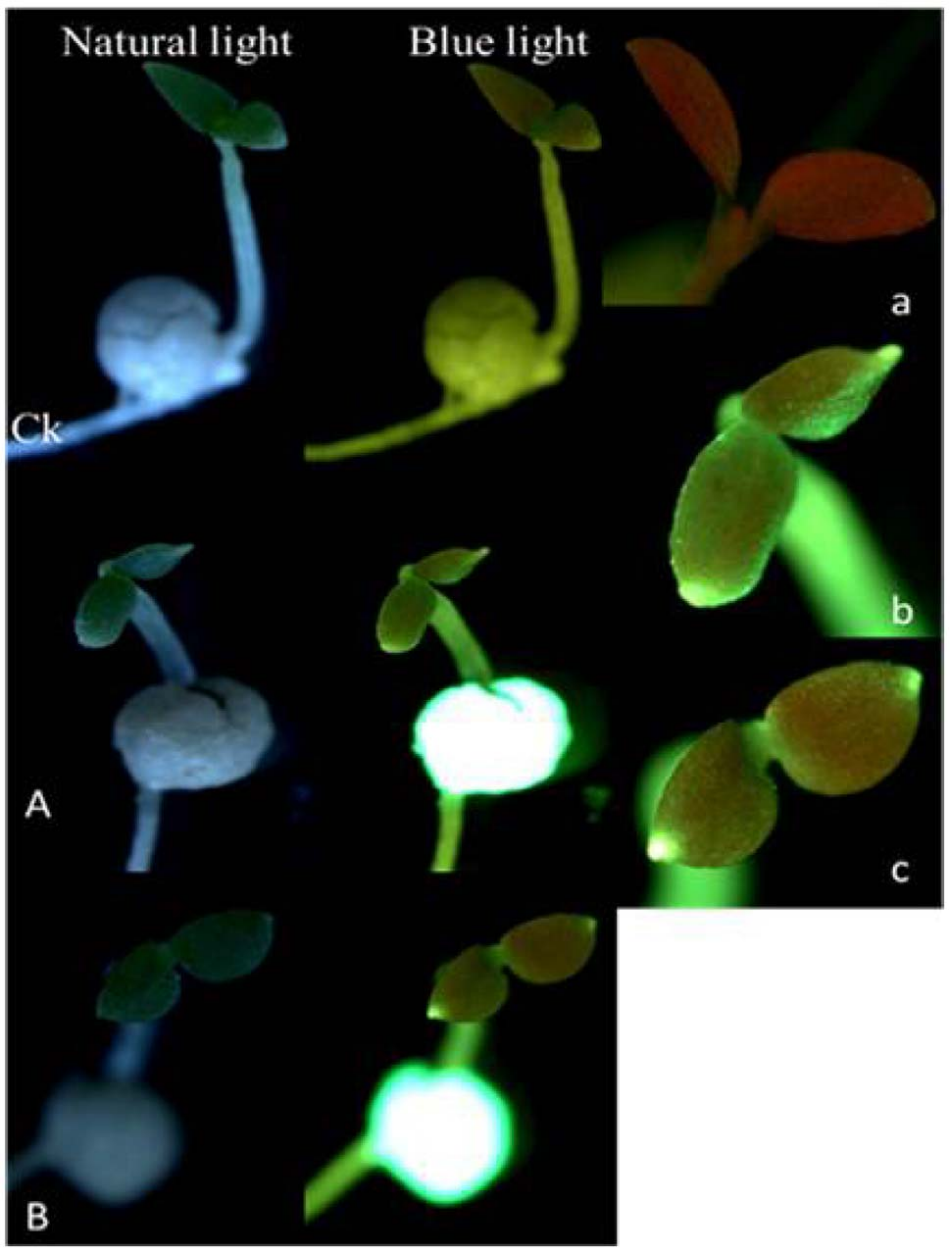
Fluorescence in seedling of MSYY85 pelleted with T1 method under illumination of different lights. **Ck**: the top row, seedlings from control seeds pelleted without fluorescein and magnetic powder after 7 days germination (×7); two columns from left to right: under natural light and blue light, respectively; **A** and **B**: seedlings from seeds pelleted with T1 (2 g seeds pelleted with 15 g bentonite and 84 g powder mixture which consisted of 79.6 g talc, 0.2 g fluorescein and 4.2 g magnetic powder (FR and MP dual-labels)) germinated for 7 days (A, the second row) and 16 days (B, the third row), respectively (×7); **a**: the cotyledon of control seedling under blue light excitation (495 nm) after 7 days germination (×20); **b** and **c**: the cotyledon of T1 seedling under blue light excitation (495 nm) after 7 and 16 days germination, respectively (×20).

As compared with the control (Figure 4Ck and 4a), the bright green fluorescence could be detected in the 7-day-old seedlings of T1 under blue light excitation (Figure 4A and 4b), and even after seedlings growing for another 9d, the green fluorescence in cotyledons tip was still extremely bright (Figure 4A and 4c). The T1 had the similar results to the T2 (data not shown).

**Figure 5:**
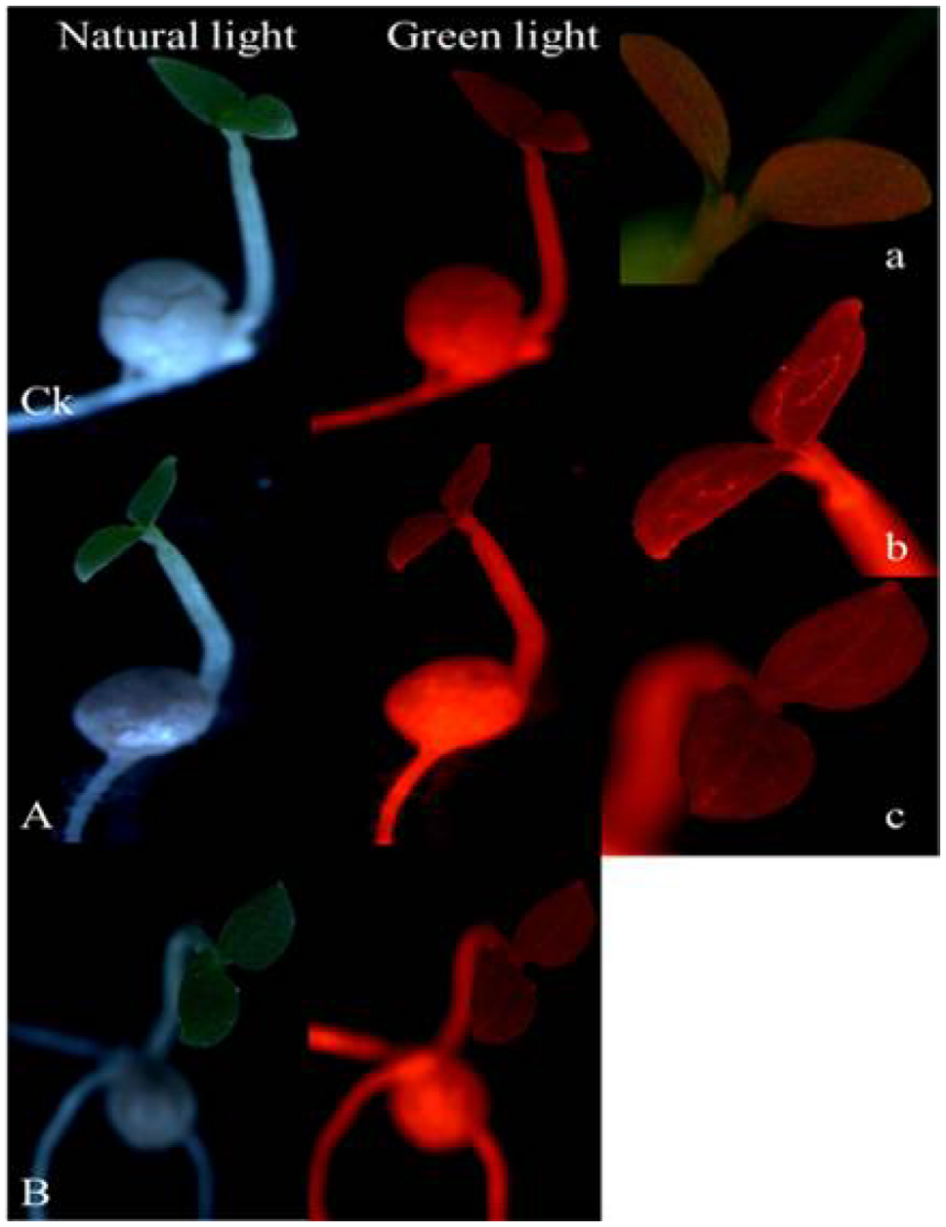
Fluorescence in seedling of MSYY85 pelleted with T3 method under illumination of different lights. **Ck**: the top row, seedlings from control seeds pelleted without rhodamine B and magnetic powder after 7 days germination (×7); two columns from left to right: under natural light and green light, respectively; **A** and **B**: seedlings from seeds pelleted with T3 (2 g seeds pelleted with 15 g bentonite and 84 g powder mixture which consisted of 79.8 g talc and 4.2 g magnetic powder. Meanwhile, a 2.0 mg/ml of rhodamine B solution was sprayed in place of water when the seed was coated with bentonite (RB and MP dual-labels)) germinated for 7 days (A, the second row) and 16 days (B, the third row), respectively (×7); **a**: the cotyledon of control seedling under green light excitation (546 nm) (×20); **b** and **c**: the cotyledon of T3 seedling under green light excitation (546 nm) after 7 and 16 days germination, respectively (×20).

After treated with T3, the cotyledons vein of 7-day-old and even 16-day-old seedlings showed obviously red fluorescence when excitation by green light (Figure 5b and 5c). However this phenomenon could not be found in the seedling of the control (Figure 5a). The T4 had the similar results to the T3 (data not shown).

**Figure 6:**
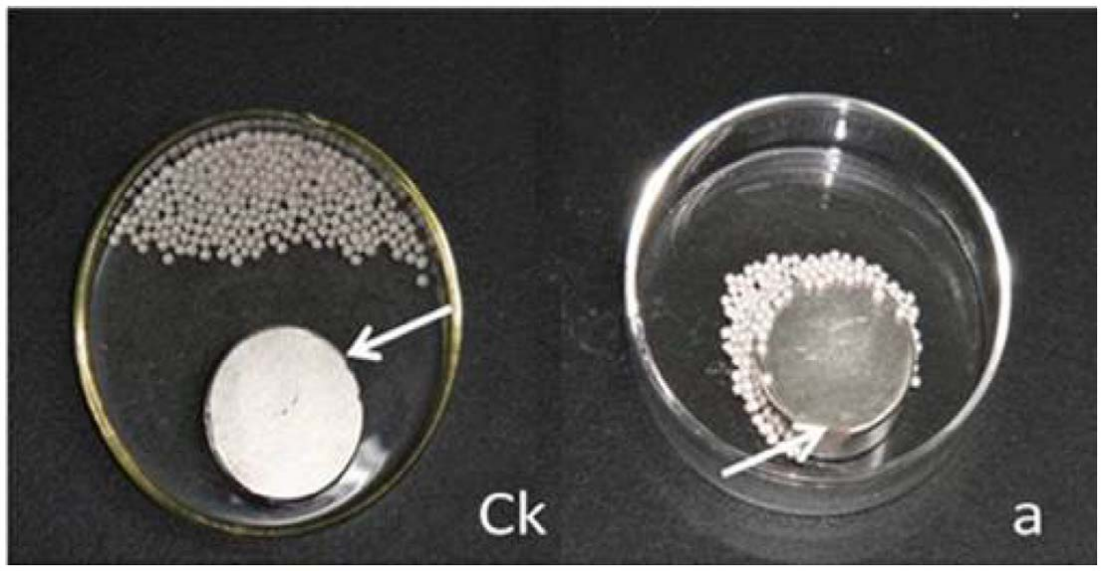
The tobacco seeds of MSYY85 pelleted with T1 method were attracted by a small magnet. **Ck**: the control seeds pelleted without magnetic powder and fluorescent materials did not be attracted by a magnet; **a**: the seeds pelleted with T1 (2 g seeds pelleted with 15 g bentonite and 84 g blend powder which consisted of 79.6 g talc, 0.2 g fluorescein and 4.2 g magnetic powder) were attracted by a magnet; the white arrow showed a small magnet.

Moreover, the pelleted seeds of T1 could be attracted by small magnet as well as the pellets of T2, T3 and T4 (Figure 6a). However, the pellets without magnetic powder could not be attracted (Figure 6Ck).

## Discussion

According to our previous results, the suitable magnetic powder concentration for seed pelleting was from 2% to 8% (the weight ratio of magnetic powder to the talc powder), and that adding magnetic powders to the second layer of pellets was the better treatment [33]. Therefore, 5% and 8% had been chosen to be two concentrations of magnetic powder in this study. Moreover, the 10∶100 (the weight ratio of FR dry powder to the naked seeds) was considered as the more suitable proportion for FR to be used on tobacco seeds [19], and the talc powder (the second layer of pellets) has less influence on the fluorescence of FR than bentonite (data not shown). In addition, that 2.0 mg/ml of RB solution was applied when seed pelleted with bentonite (the second layer of pellets) had more obvious fluorescence in seedling than talc and other RB concentrations (data not shown). Therefore, based on those single-label methods, four dual-label pelleting treatments were designed with the two selected fluorescent materials and magnetic powder in this experiment.

The better dual-label anti-counterfeiting treatments should have no negative influence on seed germination and seedling growth. And both of two labels could be detected on pellets or seedling under special device. Then they would be used as anti-counterfeiting markers. If seed is treated inappropriately, many unfortunate consequences to seed germination or seedling establishment will happen [34], such as the excessive generation of reactive oxygen species (ROS) [35]. ROS injures plant growth by damaging structure of nucleic acid, retarding protein synthesis, starting peroxidation and damaging membrane system. However, some antioxidant enzymes such as SOD could reduce these damaging effects [36]. Also, MDA is a major component of thiobarbiturate-reactive substances and its concentration often used as an indicator of lipid peroxidation in plant cells. Besides, chlorophyll and carotenoid were related closely to normal growth and development of plant [28]. The present results showed that appropriate dosage and usage of FR, RB and magnetic powder would not cause damage on normal seed germination and seedling growth, and even could improve seed establishment to some extent. The results were consistent with our previous results [19], [33].

After being treated with fluorescent compound and magnetic powder simultaneously, cracked seed pellets showed bright yellow-green or red fluorescence due to the existence of FR or RB under the illumination of blue or green light, respectively, and their characteristic of being attracted by magnet provided them dual anti-counterfeiting function. In this paper, seeds pelleted with FR and magnetic powder together showed bright yellow-green fluorescence in seedling cotyledon, especially cotyledon tip, under the illumination of blue light. However, the reason for the higher concentration of FR in the cotyledon tip than other tissues still warranted further study. The fluorescence of FR was still clearly visible in 16-day-old seedlings and its intensity was pronouncedly higher than that of the 16-day-old seedlings treated with FR alone (data not shown). In addition, the seedling treated with RB plus magnetic powder showed red fluorescence on cotyledon vein under illumination of green light. This phenomenon was also been found in tobacco seedling treated by another fluorescent dye Safranine T [25]. It suggested that the fluorescent tracer might be up-taken by seed radical and then move up to the above ground portion of seedlings after seed germinated. This speculation was consistent with the deduction of Salanenka and Taylor [37] studying the seed coat permeability and uptake of applied systemic compounds. The red fluorescence of RB was still clearly visible when seedling grew for 16 days. However, the seedling treated with RB alone had no longer red fluorescence at that time. Those results suggested that magnetic powder could slow down decaying speed of these two fluorescent labels in seedlings and increase the fluorescent intensity, to make a more lasting anti-counterfeiting effect.

For dual-label anti-counterfeiting technologies shown in this study, the fluorescent material represents a potential label to recognize rapidly and easily the true seed under special device. Meanwhile, the addition of magnetic powder enabled the pellets to be attracted by magnet, and be more suitable for precise sowing in the field and greenhouse to improve final-stand establishment and crop uniformity and to save seed and production costs. In conclusion, compared with the single-label anti-counterfeiting technology [25], the dual-label methods were undoubtedly harder to be simulated, and the two kinds of labels existed simultaneously in the same tobacco seed pellets further improved anti-counterfeiting level, enhanced the seed security and ensured tobacco production safety. In the future, the anti-counterfeiting effects of these novel dual-label methods will be verified for other crop seeds and this technology will benefit crop production from the use of real high quality seeds.
